# Medical cannabis utilization in children – a study based on a nationwide cohort

**DOI:** 10.3389/fphar.2025.1646560

**Published:** 2025-11-21

**Authors:** Nir Treves, Noa Yakirevich-Amir, Karel Allegaert, John N. van den Anker, Elkana Kohn, Maya Berlin, Ariela Hazan, Elyad Davidson, Matitiahu Berkovitch, Omer Bonne, Orit E. Stolar, Ilan Matok

**Affiliations:** 1 Division of Clinical Pharmacy, School of Pharmacy, Faculty of Medicine, The Hebrew University of Jerusalem, Jerusalem, Israel; 2 Department of Psychiatry, Hadassah Hebrew University Medical Center, Jerusalem, Israel; 3 Department of Pharmaceutical and Pharmacological Sciences, Department of Development and Regeneration KU Leuven, Leuven, Belgium; 4 Department of Hospital Pharmacy, Erasmus MC, Rotterdam, Netherlands; 5 Division of Clinical Pharmacology, Children’s National Hospital, Washington, DC, United States; 6 Departments of Pediatrics, Pharmacology & Physiology, and Genomics & Precision Medicine, George Washington University School of Medicine and Health Sciences, Washington, DC, United States; 7 Clinical Pharmacology and Toxicology Unit, Shamir Medical Center (Assaf Harofeh), Zerifin Affiliated with Faculty of Medicine, Tel-Aviv University, Tel Aviv, Israel; 8 Department of Anesthesia, Hadassah Hebrew University Medical Center, Jerusalem, Israel; 9 The Andy-Lebach Chair of Clinical Pharmacology and Toxicology, Faculty of Medicine, Tel-Aviv University, Tel Aviv, Israel; 10 Child Development Division, Maccabi Health Services, Tel Aviv, Israel; 11 Division of Clinical Pharmacy, Institute for Drug Research, School of Pharmacy and the David R. Bloom Center of Pharmacy, Faculty of Medicine, The Hebrew University of Jerusalem, Jerusalem, Israel

**Keywords:** cannabidiol, delta-9-tetrahydrocannabinol, pediatrics, developmental pharmacology, pharmacovigilance

## Abstract

**Introduction:**

This study aimed to evaluate the utilization of medical cannabis in a pediatric population and compare short-term persistence rates with those in adolescents and young adults.

**Methods:**

In this retrospective, nationwide cohort study supplemented by data from an open-label study of children with ASD, patient cases under 12 years of age who received medical cannabis treatment between 2018 and 2022 were analyzed. The primary outcome assessed was treatment persistence within the first 3 months. Secondary outcomes included changes in THC ratios, amounts dispensed, and reasons for treatment discontinuation.

**Results:**

The patient population consisted of 1,341 children using medical cannabis for ASD (751), epilepsy (330), Tourette syndrome (165), and pediatric cancer (95). Out of 3,007 consecutive medical cannabis sessions, the adjusted hazard ratio for discontinuation in the first 3 months was 0.83 (95% CI [0.71–0.96], p = 0.01) for young adults compared to children. Approximately 60%–70% of children discontinued therapy within the first 6 months. Significant alterations in THC ratios or dispensed amounts were observed in most sessions within the initial 6 months. In the open-label study dataset, most treatment discontinuations were primarily attributed to adverse effects and a perceived lack of therapeutic efficacy.

**Conclusion:**

Our findings suggest that short-term persistence of medical cannabis therapy is lower in children compared to adolescents and young adults. Moreover, many pediatric patients required adjustments to their THC ratios and showed a high frequency of treatment discontinuation. These observations underscore the importance of targeted strategies to improve medical cannabis treatment effectiveness and adherence in the pediatric population. Although MC may offer therapeutic benefits for pediatric patients, our findings emphasize the importance of careful patient selection and close medical follow-up to optimize clinical outcomes.

## Introduction

Utilization of medical cannabis (MC) has become increasingly prevalent in clinical settings in recent years (de Souza et al., 2022). The two primary cannabinoids used therapeutically are delta-9-tetrahydrocannabinol (THC) and cannabidiol (CBD). Cannabis refers to the Cannabis sativa or Cannabis indica plant and its various preparations, such as dried flowers, extracts, and edibles, all of which contain multiple bioactive components. Cannabinoids are specific compounds in cannabis that interact with the endocannabinoid system. These include phytocannabinoids like THC and CBD, as well as endogenous cannabinoids and synthetic analogues. The clinical effects of cannabis products depend on their cannabinoid composition, dosage, and method of administration (Klimkiewicz and Jasinska, 2017; Klimkiewicz and Jasinska, 2017).

Because much of the pediatric evidence focuses on CBD, it is important to note that CBD is non-intoxicating and acts through multiple targets, most notably as a negative allosteric modulator of CB1. Its safety profile has been established in randomized trials and described in the FDA label (somnolence, diarrhea, decreased appetite, and transaminase elevations; FDA-approved for LGS, DS, and TSC in patients aged ≥1 year) (Tham et al., 2019). In contrast, THC is a partial agonist at CB1 and CB2 receptors, produces psychoactive effects, and pediatric authorities recommend minimizing THC exposure in youth. Therefore, CBD-dominant products are generally preferred for cannabinoid therapy in children (Vučkovic et al., 2018).

Medical Cannabis products are currently indicated for a broad range of indications (de Souza et al., 2022), whereas in the pediatric population the use of MC is currently limited to specific indications, most common of which are ASD (Aran et al., 2021; Hacohen et al., 2022) epilepsy (Billakota et al., 2019; Thiele et al., 2019), Tourette syndrome (Barchel et al., 2024), pediatric cancer (Malach et al., 2022), and inflammatory bowel diseases (Simonian et al., 2020). In the United States, only Epidiolex is FDA-approved for Dravet and Lennox–Gastaut syndromes (FDA Epidiolex Prescribing information).

Although the therapeutic use of medical cannabis is expanding in pediatric populations, research examining its usage characteristics are still scarce.

Moreover, treatment guidelines for pediatric patients are largely derived from small randomized controlled trials, primarily focused on cohorts with refractory epilepsy syndromes (Devinsky et al., 2017; Porcari et al., 2018; Thiele et al., 2019). A recent systematic review on the use of medical cannabis in pediatric neuropsychiatric and neurodevelopmental disorders reported a low treatment dropout rate. Nevertheless, the review highlighted the limited existing data for medical cannabis treatment and the insufficient quality of evidence supporting its efficacy (Rice et al., 2024). In this retrospective study, we aim to assess the characteristics of medical cannabis use in a pediatric cohort, across four primary indications: ASD, epilepsy, Tourette syndrome, and pediatric cancer.

## Methods

This study includes a retrospective cohort analysis utilizing real-world data from the Israeli Medical Cannabis Agency (IMCA) database maintained by the Israeli Ministry of Health (IMoH) (Medical Cannabis Unit). MC in Israel is manufactured and distributed according to the Israeli medical cannabis good manufacturing practice guidelines, similar to other guidelines in the MC field. The IMCA issues an MC license for a predefined period (usually 3–12 months) and a prescription to utilize MC based on physicians’ recommendations. The IMCA database contains a comprehensive computerized dataset that documents all medical cannabis (MC) dispensing records. It includes socio-demographic information, approved MC indications, standardized MC compositions (CBD and THC concentrations), monthly and total dispensed quantities, and dispensing dates. The data is recorded in the database each time a physician prescribes MC. Access to the dataset was granted to the investigators through the Timna platform, a Big Data research infrastructure that encompasses extensive medical records maintained by the Israeli Ministry of Health. This study was approved by the Hadassah ethics committee (approval number 0526–19-HMO).

### Study population

The study population consisted of two datasets which included.

1. We used the IMCA database for this study. This database includes data on Israeli residents including children) holding an IMCA permit (‘license’) to possess and use medical cannabis during the study period, issued under Procedure 106. Licenses are granted individually to patients who meet the Israeli Ministry of Health clinical criteria and are requested/submitted by an authorized physician via the IMCA process (Israeli Medical Cannabis Unit: Licenses and Prescriptions of Cannabis Products). A dataset was extracted from the IMCA database to include all pediatric cases in Israel with a documented oil-based cannabis dispensing between April 2018 and March 2022, with a particular emphasis on children under 12 years of age.
2. Collected data included demographic characteristics (gender, age, and socio-economic status, rated on a 1–10 scale, where one is the lowest and 10 is the highest based on place of residence (Hananel et al., 2022), as well as prior psychiatric diagnoses. Additionally, the dataset contained information on medical cannabis indications and dispensation details, including dates of dispensation, quantities dispensed, and cannabis composition. In this database, the primary reason for missing values is the presence of products without Good Manufacturing Practice (GMP) certification, resulting in an official product composition not being available, which represents less than 3% of the population. Pre-specified subgroup analyses compared children, adolescents, and young adults from the same IMCA dataset to assess effect modification by developmental stage and to differentiate pediatric-specific dispensing from secular trends.
3. An additional dataset was included in the analysis. This dataset originated from an open-label study conducted at Shamir Medical Center in Israel, involving pediatric ASD patients who were treated with MC, as previously published (Stolar et al., 2022). This dataset comprised 59 children and was use to assess the reasons for treatment discontinuation and to provide insights into the causes of dose reductions during the first 6 months of therapy and in the subsequent 6 months that we saw in the IMCA dataset.

In this dataset, patients were monitored throughout their medical cannabis (MC) treatment. Study personnel recorded treatment regimens, including MC dosages, medical consultations, treatment discontinuations, and their underlying reasons. This data was documented by the study team during biweekly follow-ups over a 6-month study period. At the conclusion of the study, families were given the option to continue treatment for an additional year outside the study framework. Six months later, caregivers were contacted to determine whether the treatment had been continued. The reported adverse events were classified according to MedDRA guidelines for preferred terms and system organ class.

### Outcomes

This report assessed the patterns of MC in a pediatric cohort. Our primary study evaluated the short persistence of MC therapy. In dispensing data, adherence (how closely doses are taken as prescribed) cannot be directly observed. We therefore evaluated treatment persistence. Persistence in treatment was defined by the number of prescription refills. Persistence refers to the duration of treatment until therapy was discontinued, defined as either the end of the study period or a treatment gap of at least 120 days after the last dispensing (which includes a 90-day grace period following the month when the last cannabis was dispensed).

Short persistence was defined as persistence in the first 3 months of MC treatment in children under the age of 12. The persistence was measured across four prevalent indications: ASD, Tourette syndrome, epilepsy, and pediatric cancer, and was compared to two comparable populations: adolescents (aged 12–18 years) and young adults (aged 18–30). Cases were only included in the analysis before November 2021 to allow all patients to fulfill 3 months of treatment, avoiding right censoring.

### Secondary analysis included the following study outcomes

Long-term persistence in ASD therapy. ASD was selected as it is thought to be the most prevalent indication of MC in children, long-term persistence to the treatment was analyzed. The therapy initiation date was restricted to before February 2021 to allow all patients to fulfill 12 months of treatment, avoiding right censoring. The persistence rate in the dataset from IMCA was compared with the open-label study dataset, and the reasons for discontinuation rates were examined in the open-label study.

This report assessed patterns of medical cannabis (MC) use in a pediatric cohort. The primary analysis focused on short-term persistence with MC therapy, defined as continued use within the first 3 months of treatment among children under the age of 12. Persistence rates were evaluated across four common indications: ASD, Tourette syndrome, epilepsy, and pediatric cancer. These rates were compared with those in two older populations: adolescents (ages 12–18) and young adults (ages 18–30). Only cases initiated before November 2021 were included to ensure that all cases completed at least 3 months of treatment, thereby avoiding right censoring. In this analysis the composition of cannabis was based on the dominance nature of the product: THC dominant (for products with at least 20% more THC than CBD, *vice versa*, while for those with less than 20% difference between the two components were considered as Balanced.

The secondary analysis focused on long-term persistence in children with ASD, which is considered the most prevalent MC indication in this population. For this analysis, treatment initiation was limited to before February 2021 to allow for a full 12-month follow-up. Persistence rates in the IMCA dataset were compared with those from the open-label study dataset, and reasons for discontinuation were examined based on the open-label study data.

An additional secondary objective was performed to explore the trends and patterns of use in MC treatment. To illustrate these trends, Sankey, line, and bar diagrams were generated. These visual analyses were assessed for the following parameters, including changes in dispensed amounts, THC:CBD ratios throughout the course of therapy, and therapy discontinuation within the first 6 months across different indications. Additionally, bar charts were created for four distinct age groups to assess whether persistence rates varied according to the patients age group.

### Statistical and data analysis

As this is primarily a descriptive statistic, confounders were handled predominantly through stratification by indication and age. Where appropriate, additional confounders were adjusted using Cox regression. Variables included in the Cox regression for confounder adjustment were: indication, supplied amount, cannabis composition (THC:CBD/Balanced), socioeconomic status (SES), gender, and the use of extracts versus flowers. The Cox model assessed discontinuation after 1 month of therapy. Additionally, odds ratios for discontinuation after 3 months of therapy were calculated. Python and R programming languages were used for data processing (McKinney, 2010; Kluyver et al., 2016; Therneau, 2024). The Cox proportional model was constructed to estimate the hazard ratios of cannabis discontinuations in the short term in children, compared to adolescents and young adults, by the “Survival” package in R. The Cox model was used to consider discontinuations after 1 month of therapy. In addition, Odds ratios for discontinuation after 3 months of therapy were calculated. Results are reported with 95% confidence intervals (CI) and p-value to assess statistical significance. Sankey diagrams were created to identify visual trends with a “plotly” package adjusted to R (Interactive web-based data visualization with R, plotly, and shiny).

## Results

The IMCA comprised of 1,341 children whom were treated with MC across four leading indications: ASD, Tourette syndrome, refractory epilepsy, and pediatric cancer. The age distribution ranged from 1 to 11 years across all diagnostic groups, with the exception of the Tourette syndrome cohort, which encompassed patients aged 2–11 years. This study population included all patients who received at least one bottle of MC oil, i.e., 10 g per month (Table 1). As shown in Supplementary Table S1, in ASD, epilepsy, and Tourette syndrome, most children received high CBD products. In contrast, in pediatric cancer cases, the majority of children received balanced or high THC products.

**TABLE 1:** Demographics of the pediatric population treated with medical cannabis oil.

Indication Parameter	ASD	Epilepsy	Tourette	Pediatric cancer^a^	Total
N (% of cohort)	751 (56)	330 (25)	165 (12)	95 (7)	1,341 (100)
Sex (male %)	629 (84)	195 (59)	132 (80)	70 (74)	1,026 (77)
Age (mean (SD))	7.9 (2.1)	5.7 (2.9)	8.3 (2.2)	5.3 (3.6)	7.2 (2.7)
Socio-economic rank (mean (SD))	6.1 (2.1)	5.2 (2.2)	5.7 (2.2)	5.6 (2.4)	5.8 (2.2)
Initial MC amount (mean (SD), grams)	16.2 (7.7)	19.8 (10.8)	16.8 (10.6)	19.5 (14.8)	17.4 (9.7)

^a^ Medical cannabis in pediatric cancer was divided into 44 patients subjected to 51 different sessions for the indication of active chemotherapy, while 30 patients were subjected to 44 patients for the indication of cancer symptoms.

### Short-term persistence in MC therapy by indication and age

**FIGURE 1:**
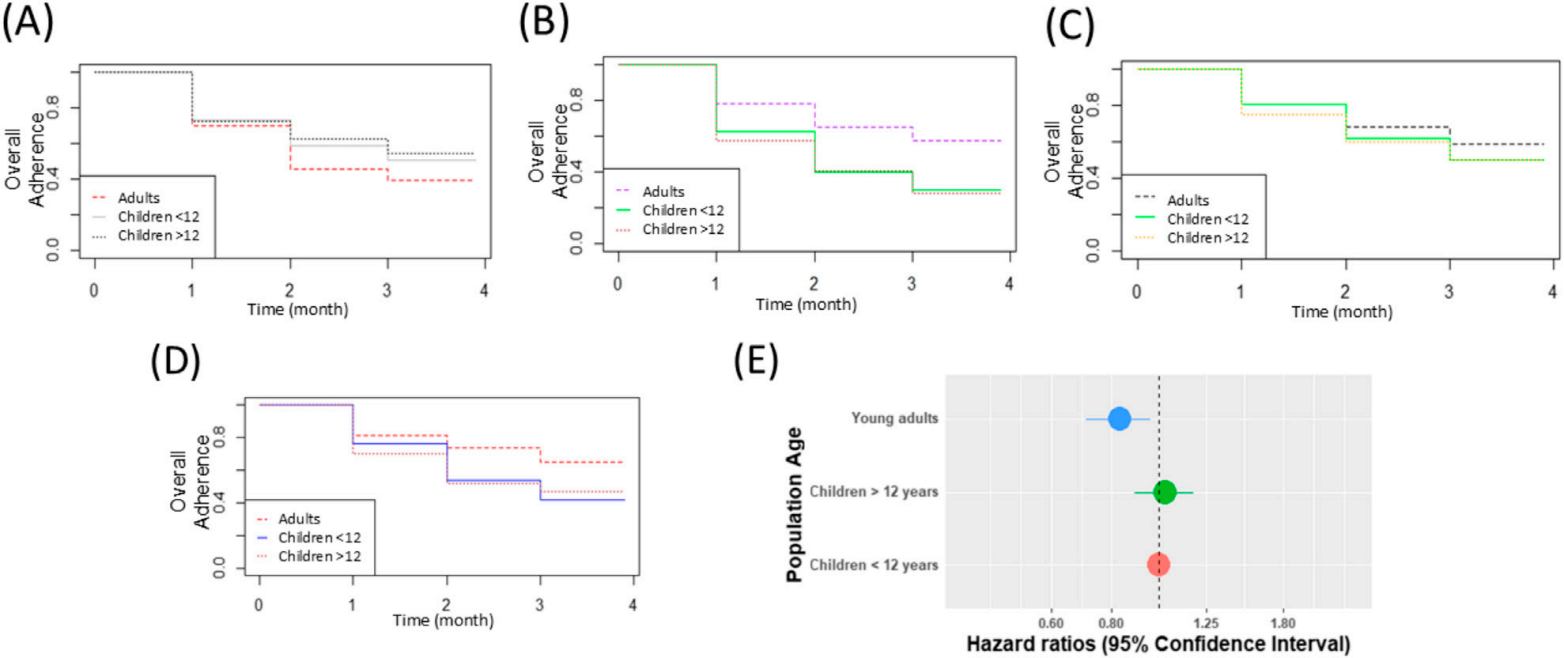
Persistence rate per indication in children below 12 years of age, compared to young adults (18–30 years) and adolescents (12–18 years) **(A)** Epilepsy **(B)** ASD **(C)** Cancer **(D)** Tourette syndrome **(E)** HR of discontinuation in adults compared to children.

Figure 1 illustrates survival curves of persistence to MC treatment in the first 3 months across four indications in children younger than 12 years old, compared to adolescents and young adults. Regardless of indications, less than 60% adhered to treatment after 3 months, and specifically in pediatric cancer and Tourette, the persistence of MC was less than 50% after 3 months of therapy.

In epilepsy, the persistence in children younger than 12 years old seems similar to the persistence in adolescents and higher than in adults (Figure 1A). In ASD, no difference was observed in persistence within the first 3 months of treatment (Figure 1B). In pediatric cancer and Tourette syndrome, adult persistence seems higher than in children (Figures 1C,D). Based on the Cox model (Figure 1E), encompassing 3,007 consecutive MC treatment sessions (A session refers to a defined period during which persistence is sustained; following any discontinuation of MC treatment, a patient may resume follow-up within a new session).

In children (1,267), adolescents (1,191), and young adults (553), the hazard ratio of discontinuations in MC treatments in adults was significantly lower than that among children (HR = 0.83, 95% CI [0.71–0.96], OR = 0.75, 95% CI [0.61,0.92]).

Supplementary Figure S1 shows the persistence of MC treatment over the first 6 months among children of different age groups, demonstrating no noticeable differences in persistence rates across the various age groups.

### Patterns of MC dispensing in children in the first 6 months of treatment

Supplementary Figures S2–S4 present Sankey diagrams showing trends in amounts and THC:CBD ratios of MC dispensed during the first 6 months of treatment across the four indications. We found, across all indications, about 60%–70% of patients discontinue treatment within 6 months.

**FIGURE 2:**
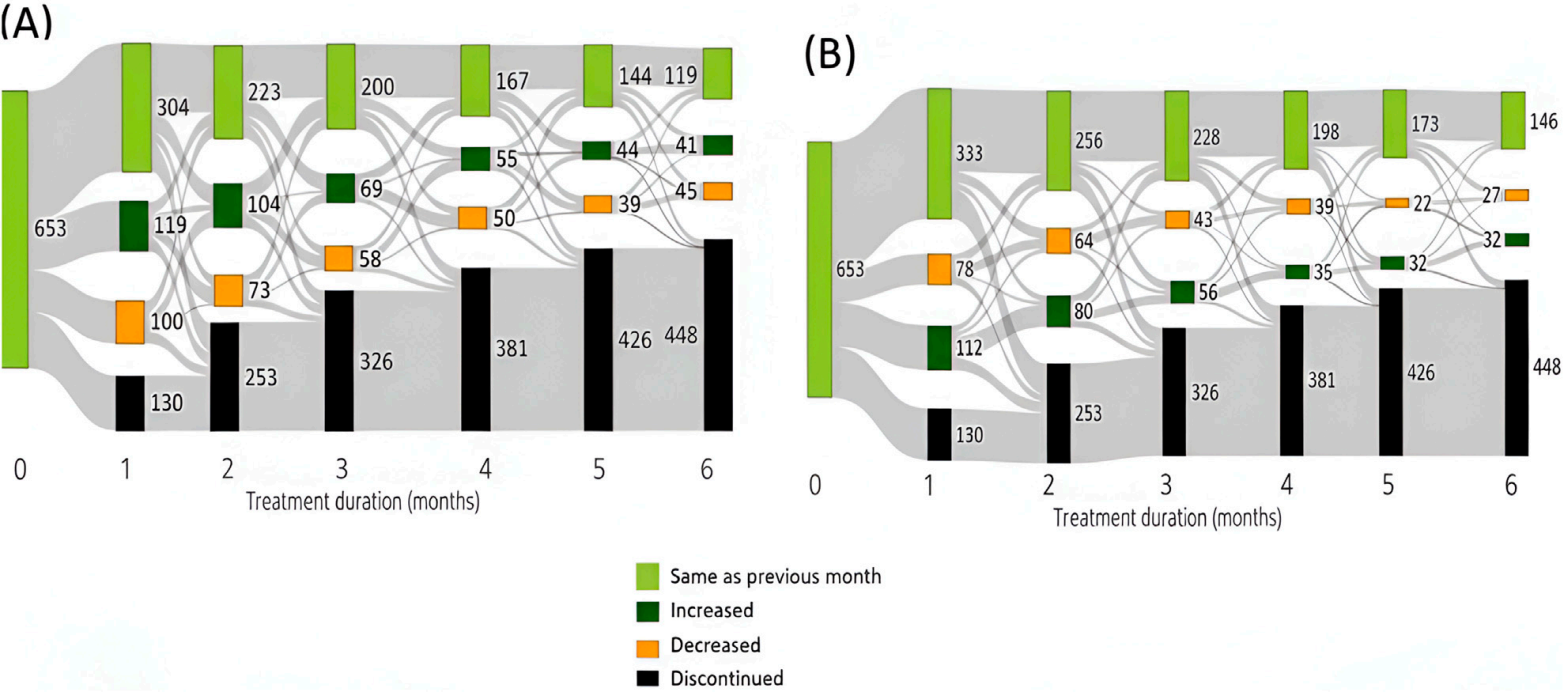
Trends in dispensing MC in the aspects of **(A)** amounts and **(B)** THC:CBD ratio among ASD pediatric population in the first 6 months of therapy in the ASD population.

As shown in Figure 2, within the ASD indication, approximately 15%–25% of patients who continued MC treatment either increased or decreased their monthly dosage during the first 6 months of therapy. Similarly, the THC:CBD ratio, about 10%–20% of these patients adjusted their ratio either increasing or decreasing it each month over the same period.

As shown in Supplementary Figure S2, whin in the epilepsy population, about 10%–20% of the patients who continued cannabis treatment increased their monthly intake of cannabis each month in the first 6 months of treatment. A small percent of these patients decreased the monthly dose of cannabis each month of treatment. Changes in the THC:CBD ratio were uncommon during the 6 months of treatment in the epilepsy population.

As shown in Supplementary Figure S3, in the Tourette population, about 15%–25% of the patients who continued cannabis treatment increased or decreased their monthly dose of cannabis each month throughout the 6 months of treatment. Changes in the THC:CBD ratio were slightly less common in that period.

As shown in Supplementary Figure S4, among the pediatric cancer population, approximately 30%–50% of patients adjusted their monthly cannabis dosage during each of the first 6 months of treatment. Likewise, changes in the THC:CBD ratio were at about 25% each month throughout the same period.

### Mean THC:CBD ratios overtime in ASD pediatric population

Supplementary Figure S5 portrays the mean THC:CBD ratio across different age groups within the pediatric ASD population over 12 months of treatment. We observed fluctuations and a gradual, continuous increase in the mean THC:CBD ratio among the older age group. In contrast, this increase was less pronounced in the other age groups, while the youngest group maintained a consistently low mean THC:CBD ratio (a low THC:CBD: commonly ≤1:10) throughout the entire treatment period.

### Dropouts and dose reductions in subgroups from open-label studies

**FIGURE 3:**
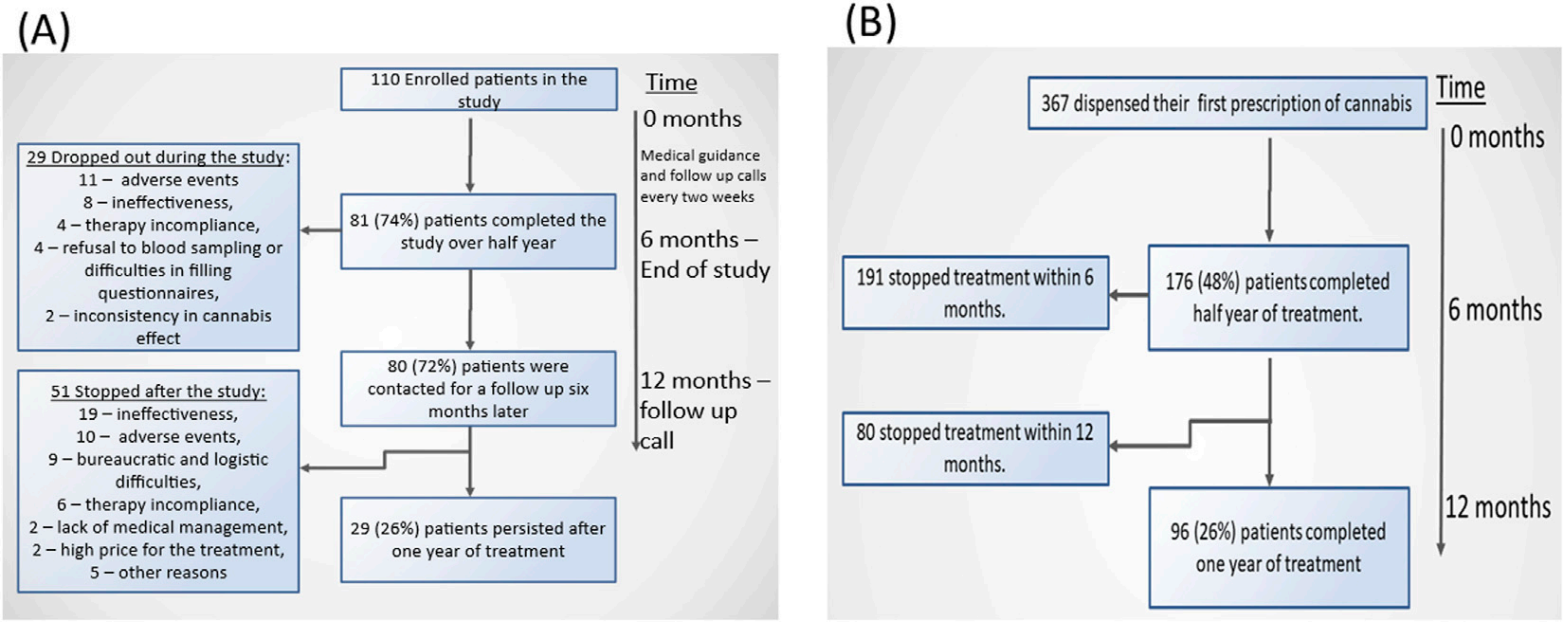
Flow charts of the persistence among the pediatric autistic populations in an open-label study **(A)** and in the retrospective cohort study **(B)**.

The flow charts in Figure 3 presents dropout rates in both study datasets. In the open-label study, approximately 75% of patients completed 6 months of therapy. The primary reasons for therapy discontinuation were adverse events and lack of treatment effectiveness. However, only 26% of the initially treated patients continued the therapy for the full year. The main reasons for discontinuation over the longer term included ineffectiveness, adverse events, and bureaucratic or logistical challenges. In the IMCA dataset, the documented completion rate for 6 months of treatment was lower, at 48%. Similarly to the open-label study, only 26% of patients continued treatment for a full year.

Table 2 presents the distribution of events associated with dose reductions in the open-label study. The majority of these reductions were chronically linked to adverse events, primarily arising from psychiatric, neurological, and general disorders.

**TABLE 2:** Causes for dose reductions in the open-label study.

Cause	Description^a^	% out of total patients (N = 52)^a^	% out of total adverse events (N = 45)
Adverse events	Psychiatric disorders, neurological disorders, and general disorders^b^	58	93
	Metabolism and nutrition disorders	8	7
Difficulties in cannabis use as indicated	Not applicable	2	Not applicable

^a^ In 33% of dose reductions, no reason was mentioned.

^b^ These reports included balance disorder, fatigue, sleep disorders, nervousness, restlessness, the feeling of despair, suicidal ideation, anxiety, nervousness, fatigue, discomfort, somnolence, obsessive-compulsive disorder, tics, aggression, psychomotor hyperactivity, general malaise, agitation, psychomotor hyperactivity, dependence, impulsive behavior, communication disorders, insomnia, crying, chills.

General disorders were included in this list as most of the adverse events under this category were related to central nervous system reactions, such as fatigue, discomfort, and crying.

^c^ One adverse event of pruritus was reported and, therefore, was not mentioned in the table.

## Discussion

To our knowledge this paper represents one of the largest real-world evidence analyses of MC use in the pediatric population. Our results indicate low treatment persistence among children, despite the use of relatively low THC doses across most indications in contrast to prior research. Previous reports in pediatric cohorts have generally shown higher retention rates, even with longer follow-up periods (Aran et al., 2019; Barchel et al., 2024). From all the cannabis products, only Epidoilox is FDA approved for Dravet and Lennox–Gastaut syndromes (FDA Epidiolex Prescribing information).

These low persistence rates may derive from specific reasons in each indication: In epilepsy, the persistence rate is the highest, reflecting MC efficacy in this indication, as shown previously in Epidiolex studies (Devinsky et al., 2017; Thiele et al., 2019). Nevertheless, there is a relatively steep dropout after the first month of treatment. Since both Epidiolex and MC are used as add-on therapies for refractory epilepsy, lack of efficacy after 1 month of treatment is unlikely to be the primary cause for the observed drop in treatment discontinuation.

In two Epidiolex clinical trials conducted in children, a few subjects suffered significant clinical deterioration, as reflected in caregivers’ global clinical impression of change. If similar outcomes occur in real-life usage, they would account for some dropouts within the first month of treatment (Treves et al., 2021).

In pediatric cancer cases, dropouts were observed during the first few months of treatment. According to IMCA guidelines, specific indications for MC treatment in the pediatric cancer population include chemotherapy-induced nausea and vomiting and pain. They can be considered for symptoms such as depressed mood and sleep disturbances (Israeli Medical Cannabis unit, n.d.). The treatment regimen typically extends over a 3–6 month period and is not confined to the chemotherapy cycles; it also includes other antiemetics, such as ondansetron, and corticosteroids. Therefore, the length of treatment sessions alone should not explain the immediate dropout cases observed. In addition, this is the only indication in which high concentrations of THC are utilized and, therefore, may indicate severe adverse events (Simonian et al., 2020; Chhabra et al., 2023).

While several studies have described the utilization of MC therapy within the pediatric oncological population, to date, no regimen protocol has been established (Ofir et al., 2019; Andradas et al., 2021; Chapman et al., 2021). A systematic review concluded that the information in online literature on MC for the pediatric cancer population is “satisfactory” and reflects a pro-cannabis opinion (Yeung et al., 2020). However, it seems that the treatment regimen and cannabis formulation are a missing theme in the available literature. This may account for the low persistence and high expectations that are not met in reality.

In children with Tourette’s and ASD, the evidence for MC efficacy is limited. The positive indications in Tourette syndrome are focused on improvement of the patients quality of life (Barchel et al., 2024), whereas in ASD, the clinical improvement is observed in the behavioral comorbidities of aggressiveness and attention deficit, with high intersubject variability (Aran et al., 2021; Hacohen et al., 2022). Previous reports within these indications suggest that most patients are not treated with conventional medicines.

Many caregivers may have high expectations for significant clinical improvement with MC, which often is not materialized. Moreover, some cases experience early although transient in many of the adverse events of restlessness and agitation, that contribute to treatment discontinuation, as in the open-label study subgroup. To address this, healthcare providers should define clearer treatment objectives and provide enhanced comprehensive guidance on MC treatment, to better align therapy expectations and improve patients’ and caregivers’ communications.

The mean THC:CBD ratio in ASD cases increased in those who adhered to the treatment regimen, particularly in the older children. We found, as could be expected, that most MC treatment sessions start with a low THC:CBD ratio, reflecting the common clinical practice (Abuhasira et al., 2019; MacCallum et al., 2021). The mean THC:CBD ratio increases over time, although the number of patients for whom their THC:CBD ratio treatment was increased was similar to those for whom their THC:CBD ratio treatment was reduced. Some physicians believe that certain patients could potentially benefit from higher doses of THC concentrations, which may explain gradual increase in THC levels of dispensed MC over time. These patients are probably also those who experienced limited response to the treatment and likely present with severe behavioral challenges. The literature about the effects of THC on the ASD population is conflicted. Although some anecdotal evidence suggests THC’s beneficial effect on behavioral disturbances in ASD, many studies linked schizophrenia, ASD, and cannabis use, exhibiting genetic and biological mechanisms in which THC may trigger psychosis-related pathways (Bortoletto and Colizzi, 2022). The findings presented here illustrate a more complex picture, indicating that THC is not necessarily avoided and possibly even utilized in some cases.

It is reasonable to speculate that avoiding exposure to higher concentrations of THC in younger children is essential, given that this period witnesses the most significant advances in brain structure and behavior (Johnson, 2001). In addition, younger children’s responses to THC may be less predictable and tolerable compared with older children, who may exhibit a similar central nervous system response to that of adolescents and adults (Donovan et al., 2016). However, our results suggested that for many pediatric cases treatment trajectories involved reductions in monthly THC:CBD ratio and amount, as opposed to “start low, go slow,” implying the occurrence of adverse events.

When comparing the two datasets at two different time points, we observed that the persistence was much higher after 6 months of treatment in the open-label study compared to real world data. However, 6 months later, when medical follow-up were comparable, the persistence declined to similarly reduced levels in both cohorts. This difference can possibly be attributed to the medical and logistical support and recruitment bias in the open-label study and highlights the contribution of medical care and follow-up to therapy. Nonetheless, the Hawthorne effect may positively affect persistence in the open-label study group during follow-up (Fernald et al., 2012).

Assuming that the subgroup analysis represents of the broader ASD population, adverse events and ineffectiveness are the most likely causes for treatment discontinuation. Dose reductions manifested in the decreased amount of dispensed MC and decreased levels of THC:CBD ratio may suggest adverse events, which was confirmed at some level in the open-label dataset, as dose reductions were associated with psychiatric and neurological adverse events.

Based on a conservative evaluation, at least 20%–30% of ASD patients suffered from adverse events that changed their therapy projections (discontinuation or therapy adjustments). In addition, lack of treatment accessibility due to lack of supply, difficulties in license renewal, or the high cost of treatment were also reasons for discontinuation. Another possible reason for dropouts is the expectations for a profound improvement in the child’s status due to the therapy (Aran et al., 2019; Hacohen et al., 2022), and corresponding disappointments when these expectations are not met.

Although the evidence generally indicates that MC treatment has limited effectiveness in ASD, many caregivers testify that their lives have completely changed positively since they started treatment (Aran et al., 2019; Hacohen et al., 2022). This discrepancy highlights the need to better understand the precise benefits of MC among the pediatric population, including patient and family-relevant variables.

This study has several limitations. Some key data points were missing, such as clear outcomes indicating the effectiveness and safety of cannabis treatment. This limitation could explain the persistence rates and usage patterns of cannabis we observed in our findings. We tried to mitigate this issue by incorporating the data retrieved from the open-label dataset; however, it was quite limited.

Additionally, it is important to note that, as this is an observational study, mainly relying on dispensing data and not on actual administration. Cannabis oil in Israel is dispensed in standardized 10 mL bottles fitted with dropper inserts. The recommended route of administration is sublingual, with patients instructed to retain the oil under the tongue–a standard procedure also mandated by the open-label study protocol. However, due to practical challenges in maintaining sublingual administration, particularly among younger children and patients with ASD, oral ingestion mixed with fatty food at room temperature is commonly employed as an alternative delivery method. The specific method of administration utilized by individual patients was not systematically documented in the present datasets, which represents a limitation of this analysis, as variations in administration route may influence bioavailability and treatment adherence. Our findings can only provide insights regarding the real-world patterns of use of MC in the pediatric population, rather than claiming any suggestions on the efficacy or safety of MC therapy in children. Moreover, due to the observational design, other biases and confounders may have influenced our results.

Furthermore, since THC and CBD are the most extensively studied and documented cannabinoid agents in medical records, this study did not explore other active cannabinoid agents, which could potentially offer more options for personalized medical cannabis therapy. However, improved characterization, medical follow-up, and providing adequate information about outcomes to parents may enhance persistence without necessitating further rigorous research. Another limitation is that we did not have access to the concomitant medications the children had, so we could not assess possible interactions of these medications with the medical cannabis. These potential interactions may have contributed to the discontinuation of treatment. We also lacked long-term follow-up data, which could have revealed different patterns of treatment persistence.

## Conclusions

Our findings demonstrated that MC treatment was frequently discontinued within a few months, primarily due to adverse effects, limited effectiveness, or logistical challenges. Among ASD patients, adverse events were the leading cause of dose reductions. In contrast, the older children tended to increase the THC exposure as treatment progressed. Despite the potential therapeutic benefits of MC for pediatric patients, our findings highlight the importance of precise patient selection and intensive medical follow-up to improve clinical outcomes. This nationwide analysis maps current patterns of pediatric medical cannabis use without making claims about clinical effects. The data we present—categorized by age, indication, and formulation—offers valuable baseline information for clinicians, policymakers, and researchers to inform the design of future studies addressing outcomes and safety.

## Data Availability

The data supporting the findings of this study are stored on the Timna platform at the Israeli Ministry of Health; however, restrictions apply to the availability of these data, which were used under license for the current study and are not publicly available. Access to raw patient data is restricted to researchers whom the institutional ethics committee has approved. Data are, however, available from the authors upon reasonable request, subject to the regulations of the Israeli Ministry of Health. Requests to access these datasets should be directed to the corresponding author.

## Abbreviations

ASD, Autism spectrum disorder; CBD, Cannabidio; CI: confidence intervals; THC, delta-9-tetrahydrocannabinol; HR, Hazard ratios; IMCA, Israeli MC Agency; IMoH, Israeli Ministry of Health; MC, Medical Cannabis; T/C, THC:CBD.
