# Supplementary material for: Medical cannabis utilization in children – a study based on a nationwide cohort
Source: Front Pharmacol. 2025 Nov 21;16:1646560. doi: 10.3389/fphar.2025.1646560 (PMC12678362; doi:10.3389/fphar.2025.1646560)
Supplement: Supplementary file 1 [file DataSheet1.pdf]

Supplementary:

Table S1 – distributions of 10-gram oil bottles in the first medical cannabis treatment dispensed per indication, divided into products.

T%C%	Autism	Epilepsy	Tourette	Pediatric cancer
T1C28	98	40	11	1
T0C24	39	35	9	3
T1C20	636	284	131	11
T3C15	202	15	22	25
T10C10	33	4	8	7
T5C10	9	2	3	3
T5C5	31	1	47	1
T20C4	16	2	4	28
T15C3	11	1	8	12
T10C2	14	2	7	38

Each row presents the components' concentrations (w/w%). T% is the concentration of THC in the preparation, and C% is the concentration of CBD in the preparation. The order of appearance was determined by the prevalence of THC concentration in the products.

Figure S1 – Persistence rate in 6 months of treatment by age groups

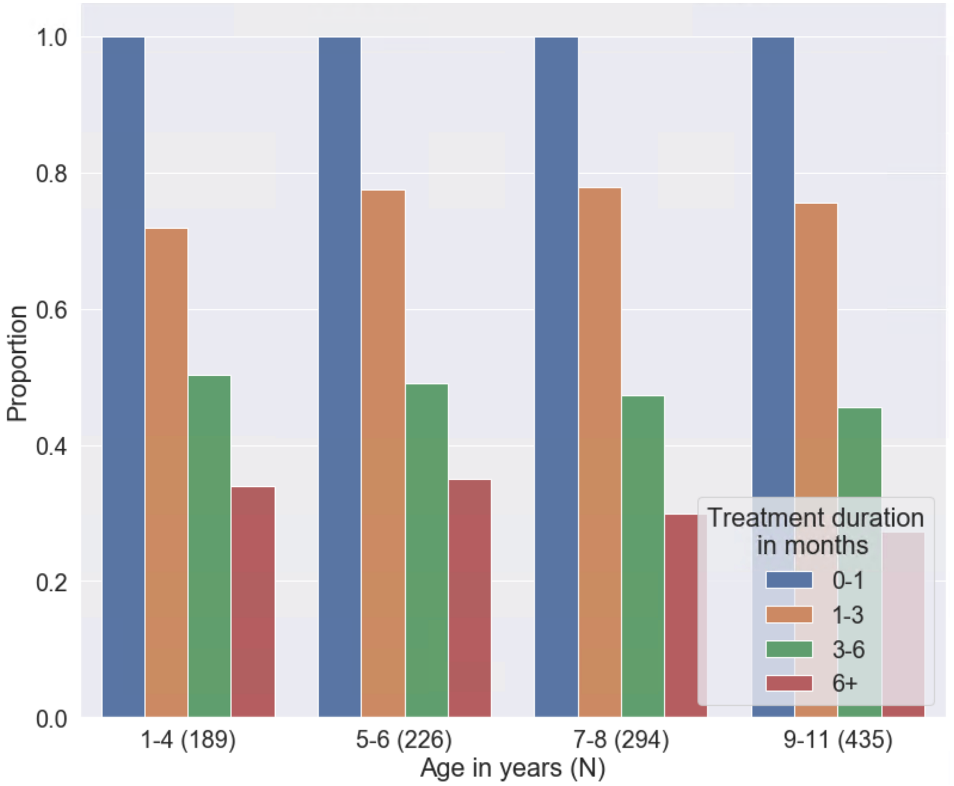

Figures S2-S4: Amounts and THC:CBD ratios in the first 6 months in epilepsy, Tourette syndrome, and oncology.

In the following figures, Black boxes indicate treatment discontinuation, dark green marks increased amount or THC:CBD ratio compared to the previous month, orange marks indicate a decreased amount or THC:CBD ratio compared to the previous month, and light green marks indicate no change in amounts or THC:CBD ratio compared to the previous month.

Figure S2a: Amount in the first 6 months of therapy in the epileptic population

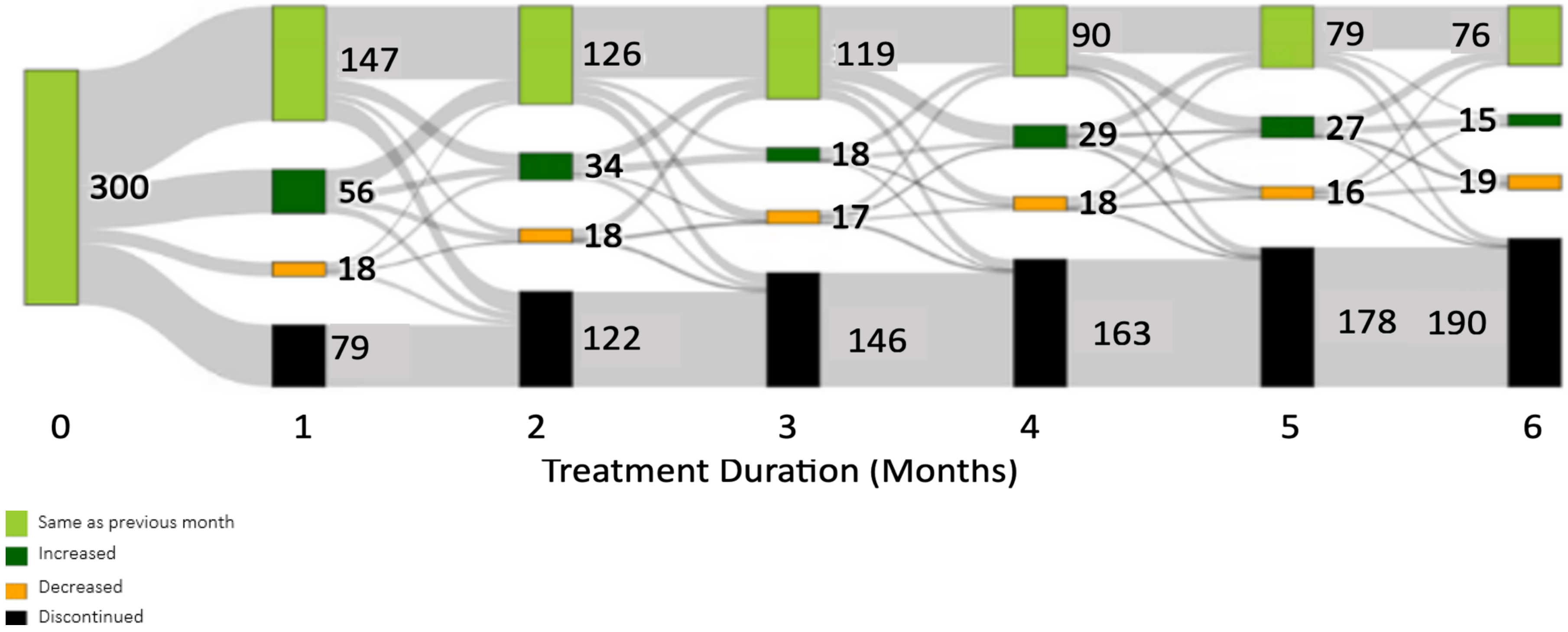

Figure S2b: THC:CBD ratio in the first 6 months of therapy in the epileptic population

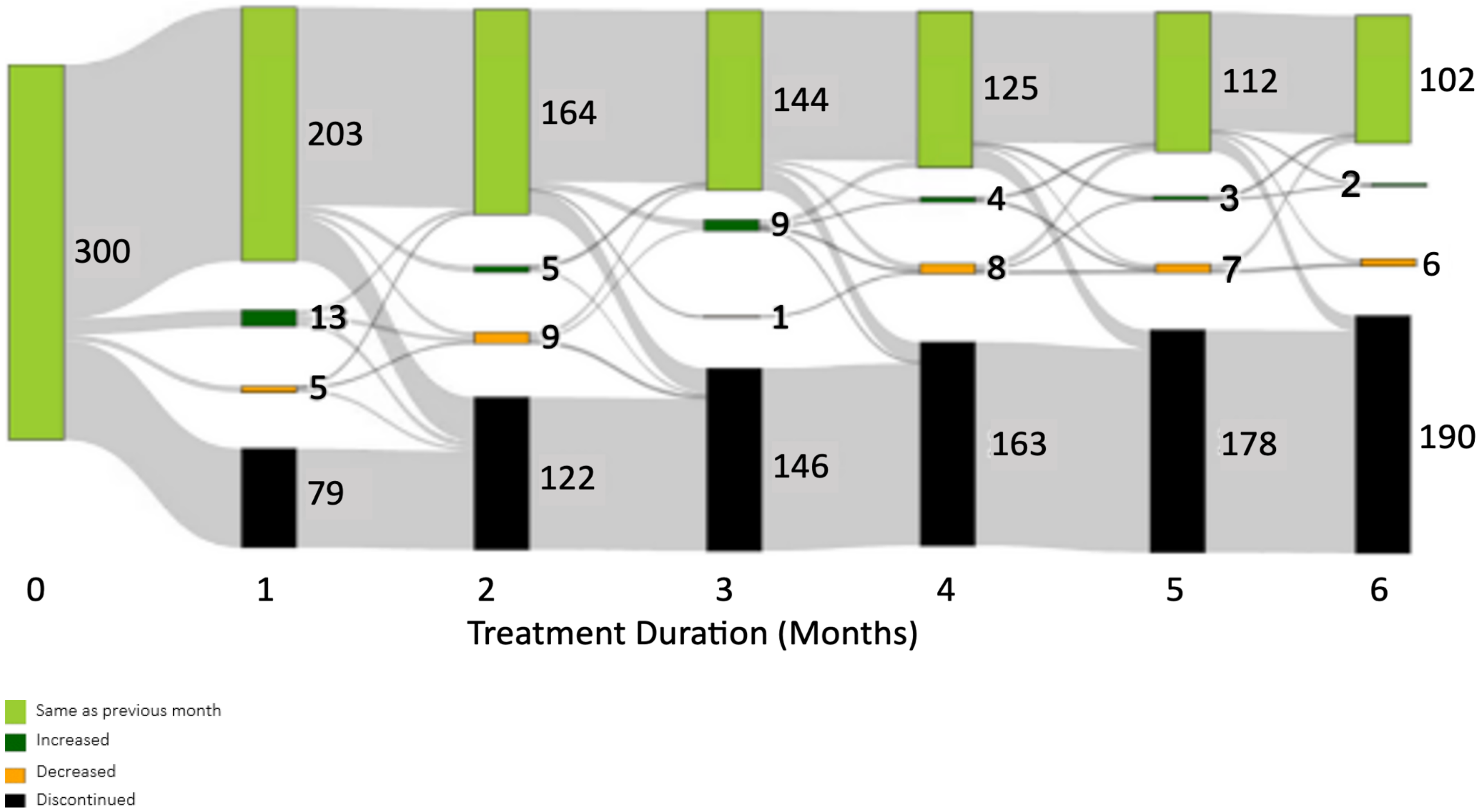

Figure S3a: Amount in the first 6 months of therapy in the Tourette population

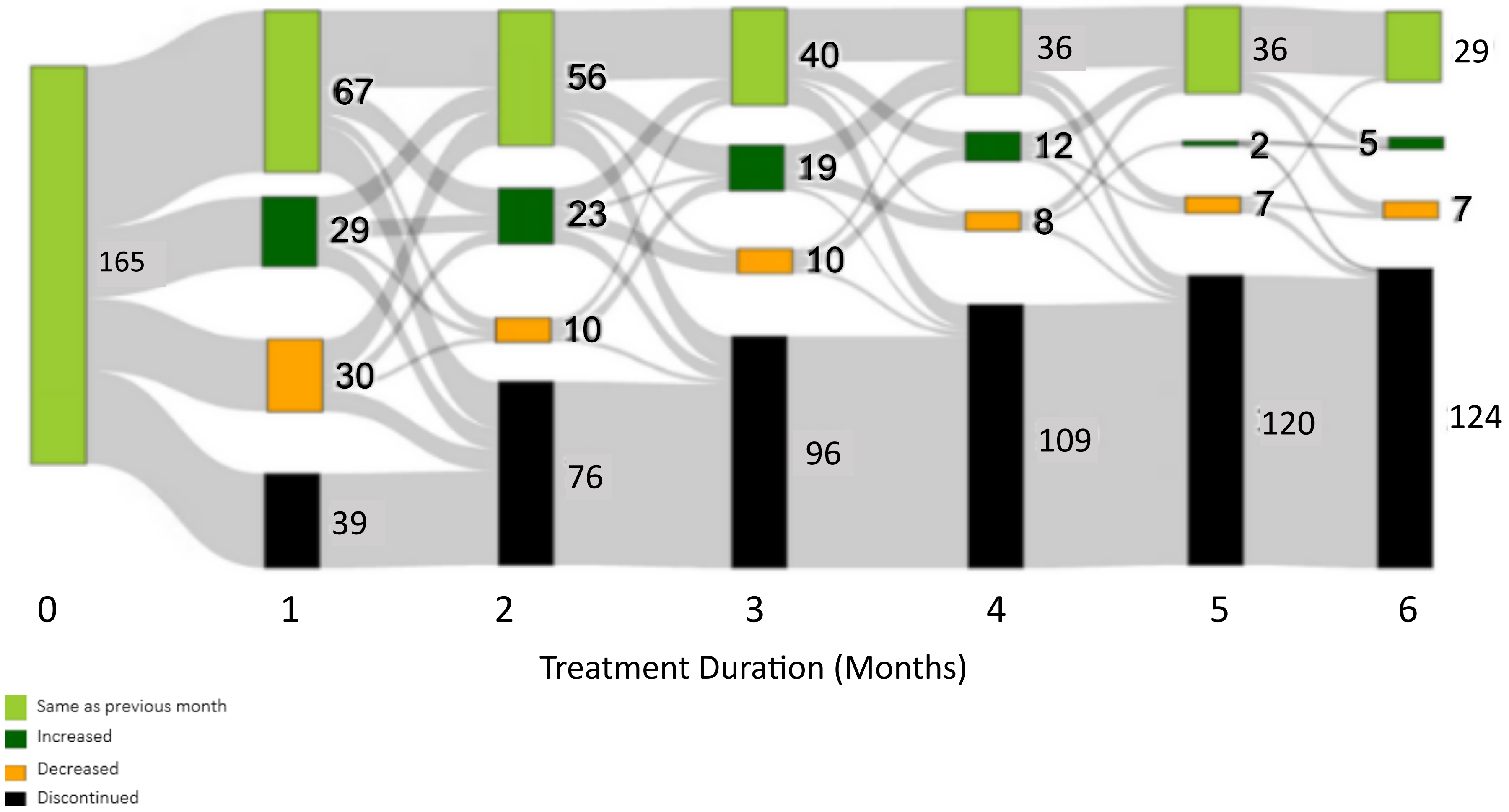

Figure S3b: THC:CBD ratio in the first 6 months of therapy in the Tourette population

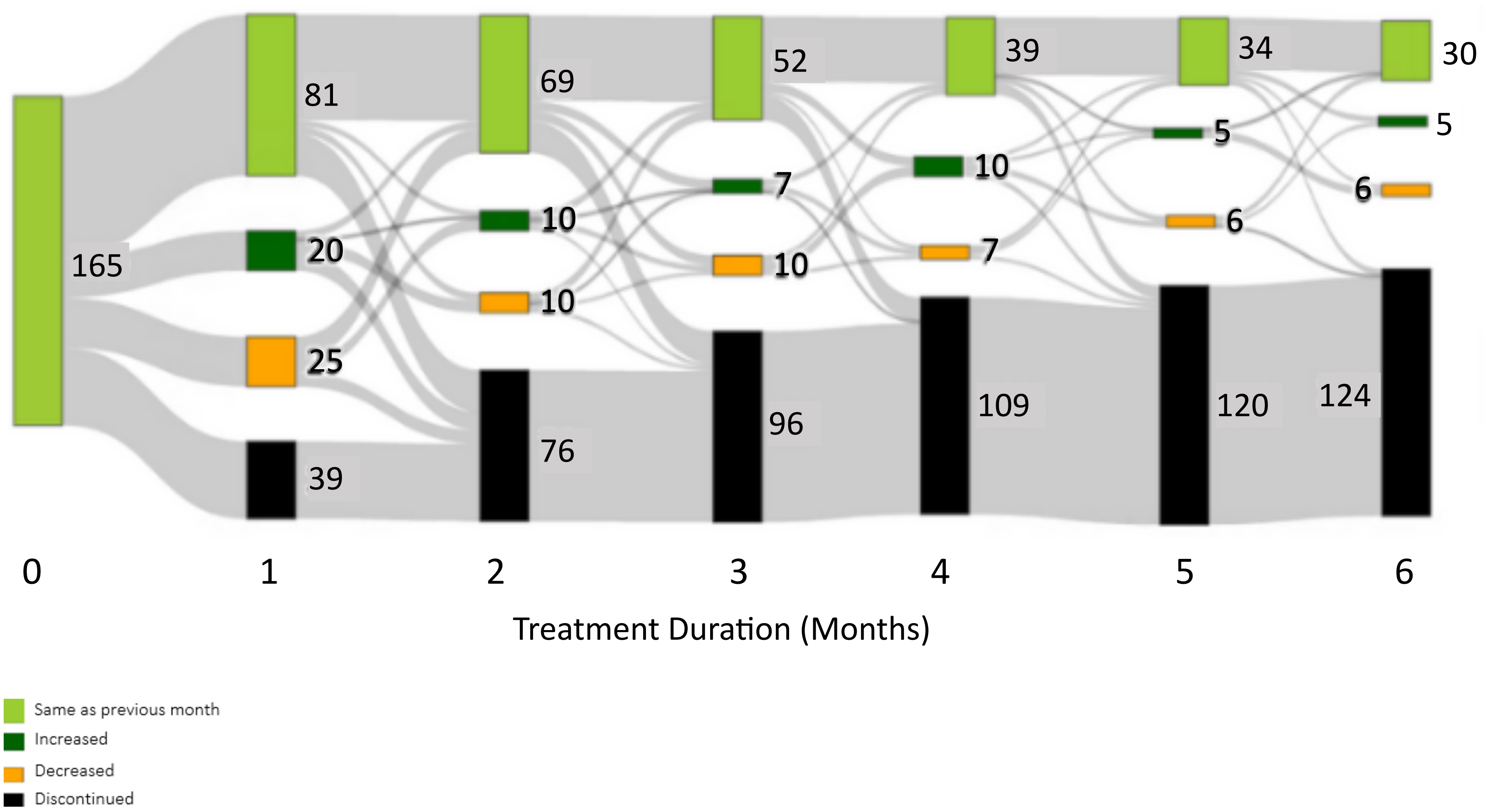

Figure S4a: Amount in the first 6 months of therapy in the oncology population

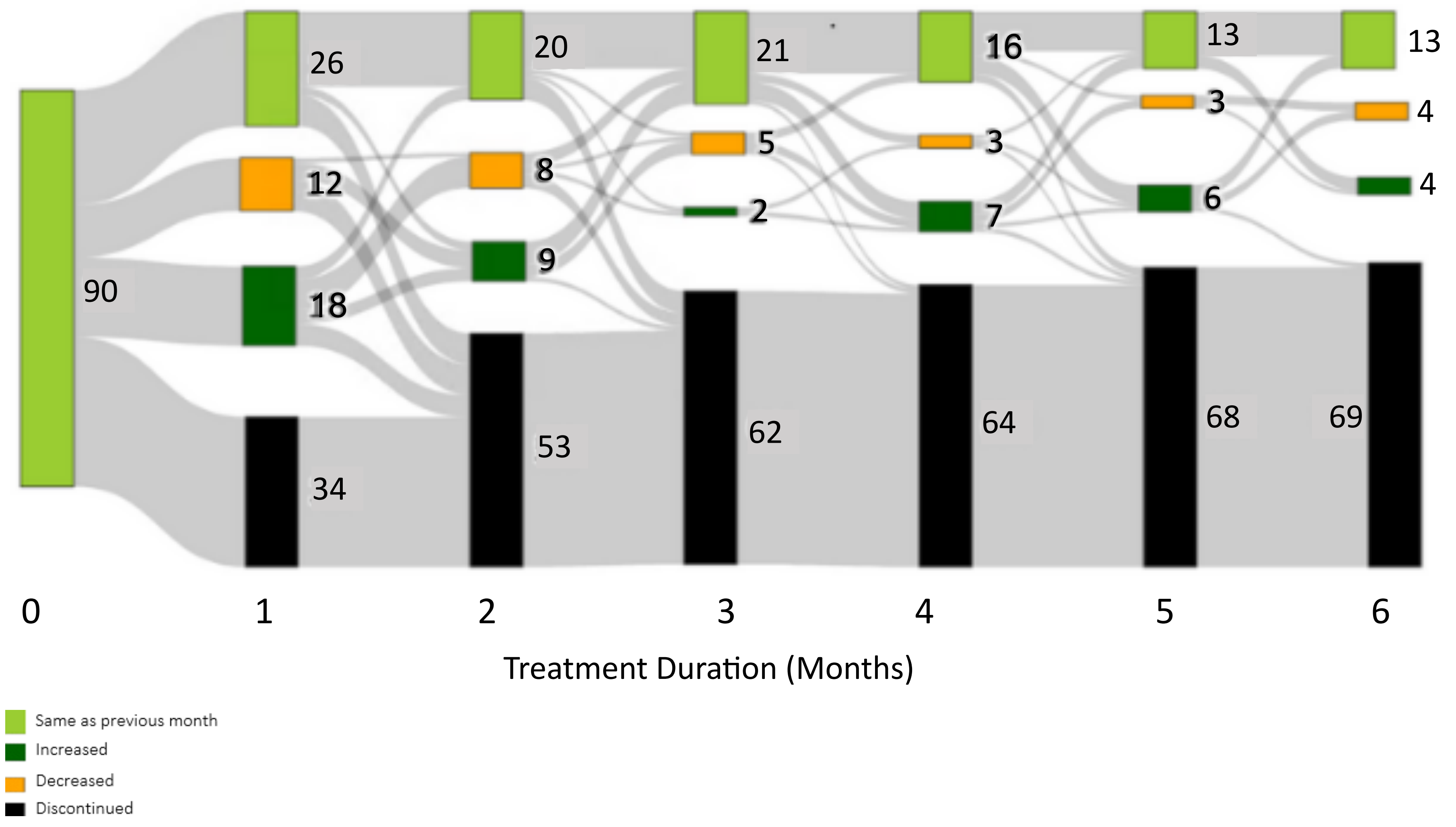

Figure S4b: THC:CBD ratio in the first 6 months of therapy in the oncology population

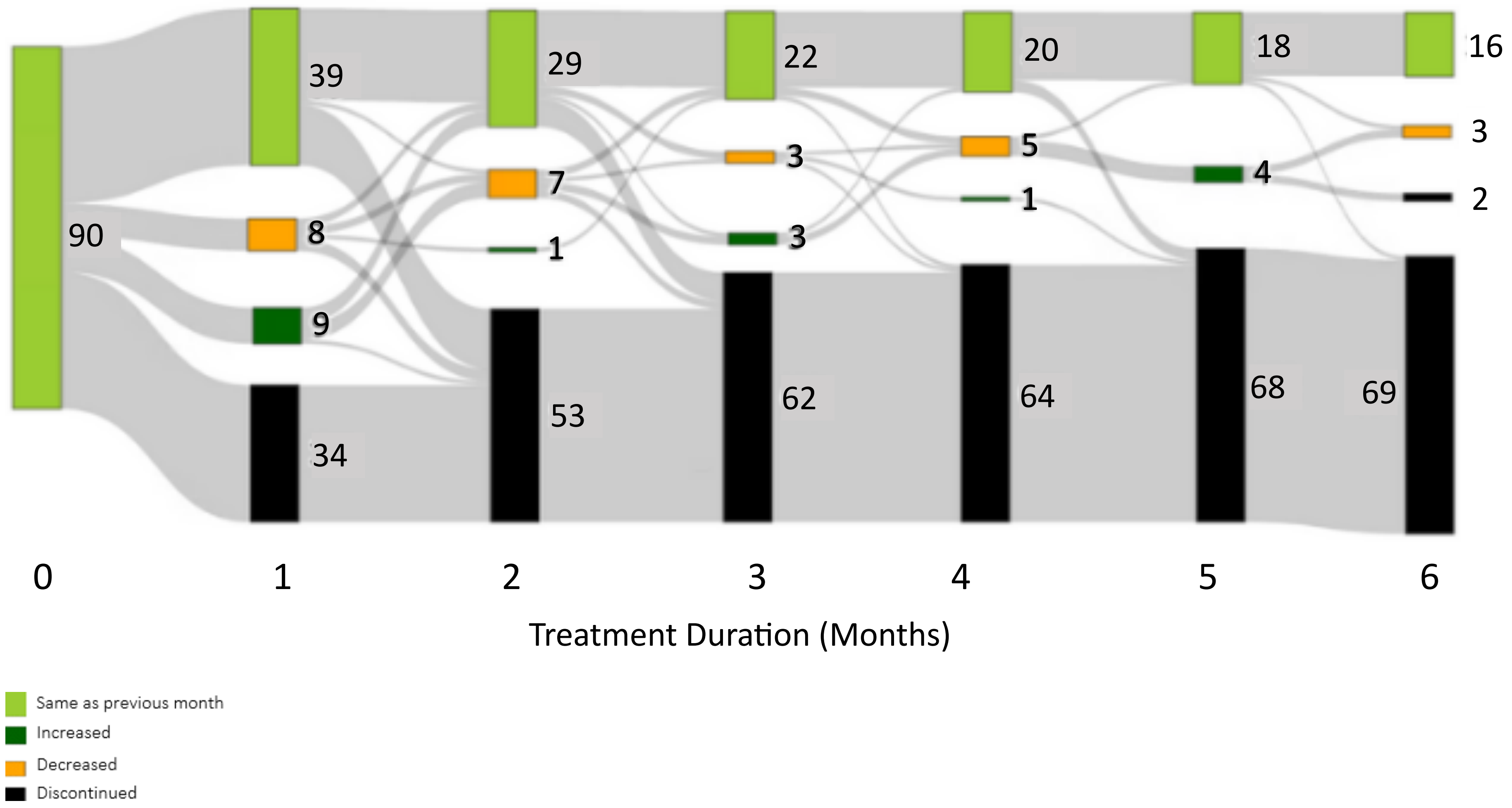

Figure S5: Mean THC:CBD ratio during treatment by age in the ASD pediatric population in the first 12 months of treatment

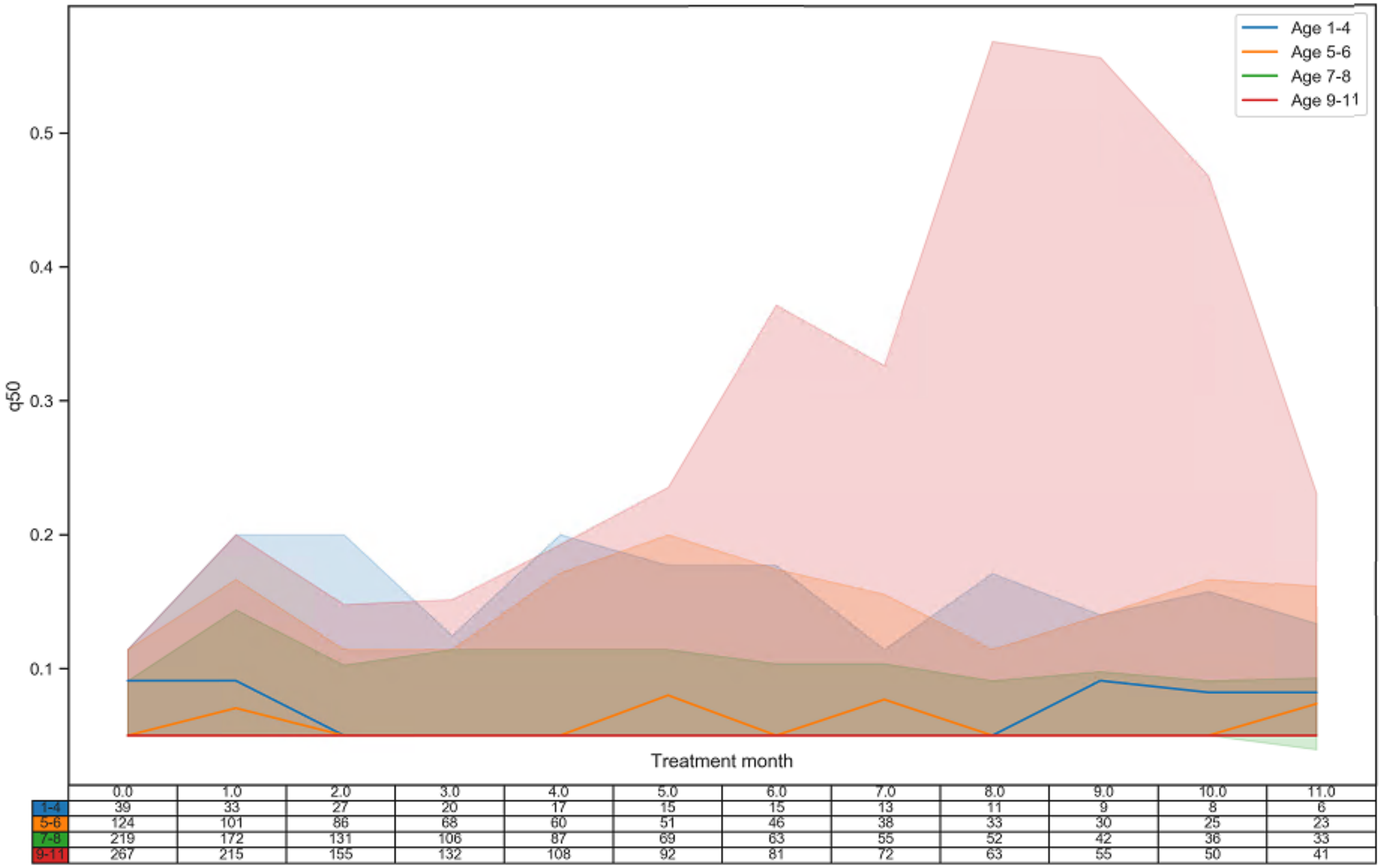

	0.0	1.0	2.0	3.0	4.0	5.0	6.0	7.0	8.0	9.0	10.0	11.0
1-4	39	33	27	20	17	15	15	13	11	9	8	6
5-6	124	101	86	68	60	51	46	38	33	30	25	23
7-8	219	172	131	106	87	69	63	55	52	42	36	33
9-11	267	215	155	132	108	92	81	72	63	55	50	41

Figure S5: Mean THC:CBD ratio with the group size that continued treatment. The shaded area under the curve is the standard deviation. Approximately 50% of the subjects have the same THC:CBD ratio.
